# Transcranial direct current stimulation combined with robotic therapy for upper and lower limb function after stroke: a systematic review and meta-analysis of randomized control trials

**DOI:** 10.1186/s12984-021-00941-0

**Published:** 2021-09-26

**Authors:** Natalia Comino-Suárez, Juan C. Moreno, Julio Gómez-Soriano, Álvaro Megía-García, Diego Serrano-Muñoz, Julian Taylor, Mónica Alcobendas-Maestro, Ángel Gil-Agudo, Antonio J. del-Ama, Juan Avendaño-Coy

**Affiliations:** 1 Neural Rehabilitation Group, Cajal Institute, Spanish National Research Council (CSIC), 28002 Madrid, Spain; 2 Toledo Physiotherapy Research Group (GIFTO), Faculty of Physiotherapy and Nursery, Castilla La Mancha University, 45071 Toledo, Spain; 3 Biomechanical and Technical Aids Unit, National Hospital for Paraplegics, SESCAM, 45071 Toledo, Spain; 4 Sensorimotor Function Group, National Hospital for Paraplegics, SESCAM, 45071 Toledo, Spain; 5 Harris Manchester College, University of Oxford, Oxford, UK; 6 Department of Physical Medicine and Rehabilitation, National Hospital for Paraplegics, 45071 Toledo, Spain; 7 Rey Juan Carlos University, 28933 Madrid, Spain

**Keywords:** Transcranial direct current stimulation, tDCS, Robotic, Neuromodulation, Stroke

## Abstract

**Background:**

Transcranial direct current stimulation (tDCS) is a non-invasive brain stimulation method able to modulate neuronal activity after stroke. The aim of this systematic review was to determine if tDCS combined with robotic therapy (RT) improves limb function after stroke when compared to RT alone.

**Methods:**

A search for randomized controlled trials (RCTs) published prior to July 15, 2021 was performed. The main outcome was function assessed with the Fugl-Meyer motor assessment for upper extremities (FM/ue) and 10-m walking test (10MWT) for the lower limbs. As secondary outcomes, strength was assessed with the Motricity Index (MI) or Medical Research Council scale (MRC), spasticity with the modified Ashworth scale (MAS), functional independence with the Barthel Index (BI), and kinematic parameters.

**Results:**

Ten studies were included for analysis (n = 368 enrolled participants). The results showed a non-significant effect for tDCS combined with RT to improve upper limb function [standardized mean difference (SMD) = − 0.12; 95% confidence interval (CI): − 0.35–0.11)]. However, a positive effect of the combined therapy was observed in the lower limb function (SMD = 0.48; 95% CI: − 0.15–1.12). Significant results favouring tDCS combined with RT were not found in strength (SMD = − 0.15; 95% CI: − 0.4–0.1), spasticity [mean difference (MD) =  − 0.15; 95% CI: − 0.8–0.5)], functional independence (MD = 2.5; 95% CI: − 1.9–6.9) or velocity of movement (SMD = 0.06; 95% CI: − 0.3–0.5) with a “moderate” or “low” recommendation level according to the GRADE guidelines.

**Conclusions:**

Current findings suggest that tDCS combined with RT does not improve upper limb function, strength, spasticity, functional independence or velocity of movement after stroke. However, tDCS may enhance the effects of RT alone for lower limb function. tDCS parameters and the stage or type of stroke injury could be crucial factors that determine the effectiveness of this therapy.

**Supplementary Information:**

The online version contains supplementary material available at 10.1186/s12984-021-00941-0.

## Background

Globally, cerebrovascular accident or stroke is a leading cause of death and disability among the adult population according to the latest estimates by the Global Burden of Disease (GBD) [1]. Most stroke patients live with disabilities affecting their quality of life, such as limb weakness or paralysis; deficits in balance, vision, or speech; and cognitive and psychological impairments [2, 3]. The rehabilitation process of these patients shows a nonlinear evolution, the clinical recovery period is shorter and the prognosis is better during the first weeks after the event (subacute phase), and the recovery is minimal or non-significant after the sixth month (chronic phase) [4–7]. For this reason, an early and intensive neurorehabilitation approach, consisting of functional and repetitive movements, should be carried out to restore normal function [8–13].

The robotic devices can provide repetitive, high-intensity and task-specific treatment of the affected limbs and measure and quantify patient progress [14]. Along with previous identified advantages, robotic therapy (RT) allows stroke survivors to perform independent training with less supervision, receive timely feedback and greater adherence to treatment [15]. However, it has been demonstrated that RT alone is not superior to other conventional rehabilitation methods, and it may be necessary to optimize its effectiveness by including complementary therapies [16].

Transcranial direct current stimulation (tDCS) is a non-invasive brain stimulation method that has been shown to be a promising neurorehabilitation intervention [17]. Its principal action mechanism is to modulate neuronal excitability networks of the affected and non-affected hemisphere after stroke through the application of low intensity direct current through superficial electrodes applied on the scalp [18]. Previous systematic reviews and meta-analyses have investigated the effects of tDCS as therapy alone or in combination with other treatments [19–22]. However, no meta-analyses have been conducted to specifically analyse the effects of tDCS as an adjunct of robotic therapy on upper and lower limb function after stroke.

The aim of this systematic review and meta-analysis was to determine whether the combined use of tDCS and robotic therapy enhances the function of the upper and lower limbs in people with stroke compared to robotic-assisted rehabilitation alone. The secondary objective was to assess the safety of tDCS and the effectiveness in combination with RT in improving strength, spasticity, functional independence and movement velocity.

## Methods

This systematic review and meta-analysis followed the protocol developed in accordance with the Preferred Reporting Items for Systematic Reviews and Meta-Analysis (PRISMA) guidelines [23] and it was registered in PROSPERO (reference number: CRD42020186963).

### Search strategy

Two independent researchers (AMG and NCS) performed an independently searched in the following databases: PubMed, Physiotherapy Evidence Database (PEDro) and the Cochrane Library. Moreover, the reference lists of all relevant articles were manually searched to identify studies that may have not been identified by the database search (Additional file 1). The databases were searched for articles published from the start of the databases until July 15th, 2021.Combinations of the following keywords were used to search the abovementioned databases: “Transcranial direct current stimulation”, “tDCS”, “non-invasive brain stimulation”, “robotic”, “robot”, “exoskeleton”, “Lokomat”, “neurological disease”, and “stroke”.

### Eligibility criteria and study selection

**Fig. 1:**
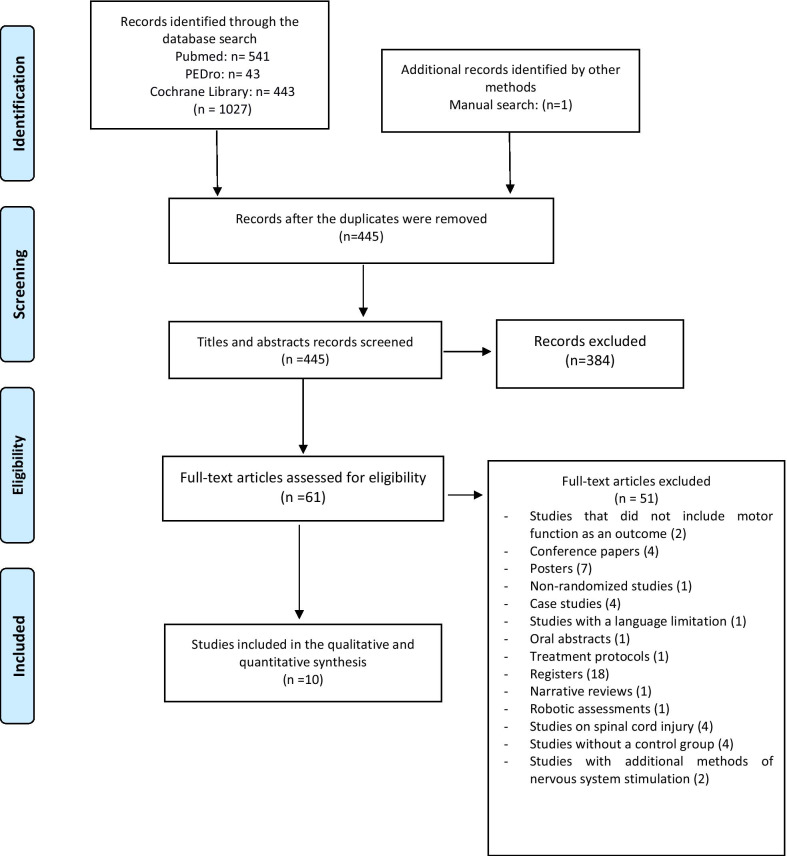
PRISMA_flow_chart

The study selection process is shown in the flowchart in Fig. 1. The studies were selected based on the PRISMA checklist’s PICOS method (P—participants; I—interventions; C—comparators; O—outcome and S—study design). We included studies in accordance with the following criteria: (1) the patients were diagnosed with a cerebrovascular accident or stroke; (2) the study was a randomized control trial (RCT); (3) transcranial direct current stimulation combined with robotic therapy was performed; (4) the intervention was compared with a control or conventional therapy; (5) the function of the upper/lower limbs was measured; and (6) the article was written in English or Spanish. Studies were excluded if they met the following criteria: (1) abstracts or congress conference papers; (2) non-human studies or preclinical trials; and (3) studies applying additional electrical stimulation as therapy. Two independent researchers (AMG and NCS) selected the studies based on the inclusion/exclusion criteria. Disagreements were resolved by consensus with a third researcher (JGS).

### Data extraction

The data were extracted by two researchers (AMC and NCS) using a chart designed for this purpose. A third researcher (JAC) compared both charts and presented the final data collected. This information was divided into two tables: Table 1, which includes basic information from the selected articles and Table 2, which includes the sociodemographic and clinical characteristics of the subjects in each study.

**Table 1:** Characteristics and design of the included studies

Author (year)	Study design	Intervention	Groups	Treatment	Parameters	Outcomes	n° sessions (s) Sessions/week (s/w)	Follow-up	Adverse effects
Geroin C. et al. (2011)^297^	RCT Double-blinded	RAGT + tDCS	G1) RAGT + tDCS G2) RAGT + sham tDCS G3) Overground-walking exercises	7 min tDCS online + 20 min RAGT + 30 leg strengthening exercise	35 cm^2^ Anode M1 affected, cathode contralateral SO 1.5 mA. CD = 0.05 mA/cm^2^	6MWT, 10MWT, SGP, Rivermead Mobility Index, MI leg, MAS	10 s 5 s/w	2 weeks	No adverse events reported
Hesse S. et al. (2011)^2830^	RCT Double-blinded	Arm robot + anodal tDCS	G1) Arm robot + anodal tDCS G2) Arm robot + cathodal tDCS G3) Arm robot + sham tDCS	20 min anodal or cathodal tDCS online + Arm Robot training	35 cm^2^ G1) Anode M1 affected, cathode contralateral SO G2) Cathode M1 unaffected, contralateral SO 2 mA. CD = 0.07 mA/cm^2^	FM/ue, MRC, MAS, BI, B&B	30 s 5 s/w	3 months	Tingling under the electrodes and transient headaches
Danz et al. (2013)^2931^	RCT Double-blinded	Anodal tDCS + Lokomat	G1) Anodal tDCS + Lokomat G2) Sham tDCS + Lokomat	20 min anodal tDCS + 20–40 min Lokomat (20–50% body support)	25 cm^2^: Anode M1 affected 35 cm^2^: Cathode contralateral SO 2 mA. CD = 0.05 mA/cm^2^	10MWT, TUG, BBS, FAC, SIS-16	12 s 3 s/w	1 months	No adverse events reported
Triccas et al. (2015)^3032^	RCT Double-blinded	Anodal tDCS + RT	G1) Anodal tDCS + RT G2) Sham tDCS + RT	20 min Anode tDCS online + 1 h Armeo robotic training	35 cm^2^ Anode M1 affected Cathode contralateral SO 1 mA. CD = 0.035 mA/cm^2^	FM/ue, ARAT, MAL-28, SIS	18 s 2-3 s/w	3 months	tDCS sensation: itching, tingling, warmth, burning, pain, light flashes and headaches RT: fatigue, shoulder pain and upper trapezius pain on the affected side
Straudi S et al. (2016)^3133^	RCT Double-blinded	tDCS + upper extremity robot-assisted	G1) tDCS + robot-assisted G2) Sham tDCS + robot-assisted	30 min tDCS + 30 min upper extremity robot-assisted	35 cm^2^ Anode M1 affected, cathode M1 contralateral hemisphere, 1 mA. CD = 0.035 mA/cm^2^	FM/ue, B&B MAL-14	10 s, 5 s/w	Immediately Post-intervention	Skin redness, under the site of stimulation (5/1) Headache (1/1) Sleepiness (1/0) Neck pain (0/1) (tDCS/sham-tDCS)
Seo HG et al (2017)^3234^	Pilot RCT, double-blinded	tDCS + RAGT	G1) tDCS + RAGT G2) Sham + RAGT	20 min de tDCS + 45 min RAGT	35 cm^2^ Anode: M1 (leg area) affected. Cathode: contralateral SO 2 mA. CD = 0.07 mA/cm^2^	FAC, 10MWT, 6MWT, BBS, FMA-LE, MRC EMG activity: MEP	10 s 5 s/w	4 weeks	No mention
Mazzoleni S, et al. (2017)^3335^	RCT Single blind	tDCS + wrist RT	G1) tDCS + robot-aided G2) Sham + robot aided	20 min tDCS online + 30 min RT	35 cm^2^ Anode: M1 (hand area) affected. Cathode contralateral orbit 2 mA. CD = 0.07 mA/cm^2^	(FM/ue, FM/w), MAS, MI, B&B Kinematic data	30 s 5 s/w	Immediately Post-intervention	No adverse events reported
Dehem et al. (2018)^3436^	RCT Double blind crossover	robot upper limb + real tDCS	G1) robot upper limb + real tDCS G2) robot upper limb + Sham tDCS	20 min tDCS/sham online + RT	35 cm^2^ Anode M1 affected, Cathode M1 unaffected 1 mA. CD = 0.035 mA/cm2	B&B, PPT Upper limb kinematics	2 s 1 s/w	Immediately Post-intervention	No adverse events reported
Edwards et al. (2019)^3537^	RCT Double blind	tDCS + Robot	G1)Sham + Robot G2) tDCS + Robot	20 min tDCS/sham + 1 h RT	35 cm^2^ Anode M1 affected (5 cm lateral to the vertex) Cathode: contralateral SO 2 mA. CD = 0.07 mA/cm^2^	FM/ue, Wolf Motor Function Test, BI, MRC, SIS, EMG activity: MEP	36 s 3 s/w	6 months	Headache, burning sensation, sleepiness, tingling, redness, trouble concentrating
Mazzoleni et al. (2019)^3638^	RCT Single blind	tDCS + wrist robot-assisted training	G1) Sham + robot G2) tDCS + robot	20 min tDCS/sham online + 30 min wrist robot-assisted training	35 cm^2^ Anode: M1 affected Cathode: contralateral orbit bone 2 mA. CD = 0.07 mA/cm^2^	FM/ue, FM/w MAS/w, MI, B&B. Wrist kinematic parameters	30 s 5 s/w	Immediately Post-intervention	No adverse event reported

Randomized controlled trial (RCT). Robot-assisted gait training (RAGT). Transcranial direct current stimulation (tDCS). Group (G). Supraorbital (SO). 6-min walk test (6MWT). 10 min walking test (10MWT). Spatiotemporal gait parameters (SGP). Motricity Index (MI). Modified Ashworth scale (MAS). Fugl-Meyer assessment scale for upper extremities (FM/ue). Barthel Index (BI). Box & block test (B&B). Medical Research Council sum score (MRC). Timed up-and-go test (TUG test). Berg balance scale (BBS). Functional ambulation category (FAC). Stroke impact scale 16 (SIS-16). Robot therapy (RT). Motor activity log 14 items (MAL-14). Action research arm test (ARAT). Motor activity log-28 (MAL-28). Milliamps (mA). Fugl-Meyer assessment scale for wrist (FM/w). Purdue pegboard test (PPT). Electromyography (EMG). Motor-Evoked Potentials (MEP). Modified Ashworth scale on the wrist (MAS/w)

**Table 2:** Sociodemographic and clinical characteristics of the subjects with stroke

Author (year)	Participants (n) [enrolled]	Gender M/F	Age Mean (SD)	Classification	Type I/H	Pathology characteristics			Duration Mean (SD)
						Affected side		Right/left	Location of lesion C/SC
Geroin C. et al. (2011)^29^	n = 30 tDCS (n = 10) Sham (n = 10) Control (n = 10)	tDCS: 8/2 Sham: 6/4 Control: 9/1	tDCS: 63.6 (6.7) Sham: 63.3 (6.4) Control: 61.1 (6.3)	Chronic	**–**	**–**		tDCS: 4/3 Sham: 5/2 Control: 3/4	tDCS:25.7 (6.0) Sham: 26.7 (5.1) Control: 26.9 (5.8) *months*
Hesse S. et al. (2011)^2830^	n = 96 Anodal tDCS (n = 32) Cathodal tDCS (n = 32) Sham (n = 32)	Anodal tDCS: 20/12 Cathodal tDCS: 18/14 Sham: 21/11	Anodal tDCS: 63.9 (19.5) Cathodal tDCS: 65.4 (8.6) Sham: 65.6 (10.3)	Subacute	Anodal tDCS: 32/0 Cathodal tDCS: 32/0 Sham: 32/0	Anodal tDCS: 14/18 Cathodal tDCS: 15/17 Sham: 16/16		Anodal tDCS: 25/7 Cathodal tDCS: 24/8 Sham: 26/6	Anodal tDCS: 3.4 ± 1.8 Cathodal tDCS: 3.8 ± 1.4 Sham: 3.8 ± 1.5 *weeks*
Danz M. et al. (2013)^2931^	n = 8 tDCS (n = 4) Sham (n = 4)	tDCS: 3/1 Sham: 1/3	tDCS: 64.75 (14.86) Sham: 70.75 (11.14)	Chronic > 12 months	tDCS: 2/2 Sham: 4/0	tDCS: 0/4 Sham: 0/4		**–**	tDCS: 4.78 (4.6) Sham: 3.22 (2.73) *years*
Triccas T. et al. (2015)^3032^	n = 23 tDCS (n = 12) Sham (n = 11)	tDCS: 7/5 Sham: 7/4	tDCS: 62.5 (14.3) Sham: 64.3 (9.94)	Subacute and chronic > 2 months	tDCS: 9/3 Sham: 9/2	tDCS: 11/2 Sham: 11/0		tDCS: 3/8 Sham:4/6	19.6 (25.7) months
Straudi. S et al (2016)^3133^	n = 23 tDCS (n = 12) Sham (n = 11)	tDCS: 5/7 Sham: 7/4	tDCS: 52.7 (16.0) Sham: 64.3 (9.7)	Subacute and chronic	tDCS: 10/2 Sham: 9/2	tDCS: 3/9 Sham: 5/6		tDCS: 9/3 Sham: 5/6	tDSC: 40.7 (35.1) Sham: 78.2 (61.9) *weeks*
Seo HG et al (2017)^3234^	n = 21 tDCS (n = 11) Sham (n = 10)	Sham: 7/3 tDCS: 9/2	Sham: 62.9 ± 8.9 tDCS: 61.1 ± 8.9	Chronic	Sham: 7/3 tDCS: 9/2	Sham: 8/2 tDCS: 5/6		**–**	Sham: 152.5 ± 122.8 tDCS: 75.5 ± 83.4 *months*
Mazzoleni S et al (2017)^3335^	n = 24 tDCS (n = 12) Sham (n = 12)	Sham: 6/6 tDCS: 1/11	Sham: 75.25 ± 8.01 tDCS: 70.00 ± 12.80	Subacute	Sham: 11/1 tDCS: 7/5	Sham: 6/6 tDCS: 6/6		**–**	Sham: 24.17 ± 14.02 tDCS:26.58 ± 11.86 *days*
Dehem et al (2018)^3436^	n = 21 Crossover	15/6	Total: 60.5 (9.5)	Chronic	Total: 15/6	Total: 11/10		14/7	Total: 38.6 (57.0) *months*
Edwards et al (2019)^3537^	n = 82 Robot + tDCS (n = 41) Robot + sham (n = 41)	50/32	67.8	Chronic	Ischaemic	Right (82)		**–**	1317 days 3.6 years
Mazzoleni et al (2019)^3638^	n = 40 tDCS (n = 20) Sham (n = 20)	Sham: 7/12 tDCS: 8/12	Sham: 68.74 ± 15.83 tDCS: 67.50 ± 16.30	Subacute Stroke	Sham: 16/3 tDCS: 13/7	Sham: 11/8 tDCS: 11/9		**–**	25 ± 7 days

Male (M); Female (F); Standard Deviation (SD); Ischaemic (I); Haemorrhagic (H); Cortical (C); Subcortical (SC); Group (G); Transcranial direct current stimulation (tDCS)

Regarding the primary outcome, we analysed the effect of the combined therapy on limb function using scales and functional tests. According to our previous protocol, when a study included more than one functional scale for upper limbs, the Fugl–Meyer motor assessment of the upper extremities (FM/ue) was considered first, which is a scale designed to assess reflex activity, movement control and muscle strength in the upper limbs [24]. For the lower limbs, the 10-m walking test (10MWT) was chosen preferably. During this test, the subject had to walk a distance of 10 m as quickly as possible [25].

The secondary variables adverse effects and patients lost to follow-up were measured as the number of participants who suffered adverse effects within each group and the number of participants lost to follow-up. In addition, we analysed the strength using the Motricity Index (MI) or the Medical Research Council scale (MRC); spasticity with the modified Ashworth scale (MAS); functional independence with the Barthel Index (BI); and kinematic parameters were assessed. Regarding the kinematics, only the velocity of movement data (degrees per second or cm per second) could be extracted. To measure the intervention effect, post-intervention scores instead of change scores were chosen. In the studies where it was necessary to obtain or clarify missing data, the corresponding authors were contacted for additional information. Data that were only represented by graphs were extracted using Graph Grabber v 2.0.1 software for graph digitalization (https://www.quintessa.org/software/).

### Risk of bias assessment

The potential risk of bias was assessed on the basis of Cochrane Collaboration’s guidelines [26]. This questionnaire was performed by two independent reviewers (MAM and DSM). Disagreements were resolved by a third senior researcher (JGS). Review Manager (RevMan) software (computer program, version 5.3, Copenhagen: The Nordic Cochrane Centre, The Cochrane Collaboration, 2014) was used to perform the analysis. Six items were addressed, and the relevant risk was expressed as three levels (unclear, low, and high). The researchers agreed prior to the assessment that for the item “blinding of the participants and personnel”, the level of risk would be rated as unclear when the participants or personnel were not blinded, and for the item “selective reporting”, studies without a registered protocol would be qualified as having unclear or high risk, depending on the final report. Additionally, funnel plots for the main variable (function) were assessed to evaluate publication bias.

### Data synthesis and analysis

The inverse variance method and a fixed-effects model were used for the 7 assessed variables. The standardized mean difference (SMD) was used to express the results for upper and lower limb function, strength and movement velocity since these variables are sometimes reported with different scales or units. Lower limb function was assessed by the 10MWT, which measures the time an individual takes to walk 10 m. A higher score indicates more severe disability, so this value was multiplied by − 1 to align the effect direction. The mean difference (MD) was used for the spasticity and functional independence results, which were expressed on the same scale in the included studies. The risk difference (RD) was calculated for the adverse events and loss to follow-up variables. The 95% confidence interval (95% CI) were calculated for all outcomes. Statistical heterogeneity was evaluated using the chi-squared test (with statistical significance set at p < 0.10) and was measured by calculating the I^2^, with values of 25%, 50%, and 75% representing low, moderate, and high heterogeneity, respectively [27].

The results corresponding to the longest follow-up period were analysed for each of the included studies. In the studies where the results were reported by the intention-to-treat and by protocol, the data from the intention-to-treat analysis were used. If participant data were available and the authors did not present the intention-to-treat results, this analysis was also performed. In the three-arm studies, the shared group was split according to the Cochrane Group guidelines [26] to avoid data being counted twice*.* In addition to the global analysis, an analysis by subgroups was conducted for the main variable, limb function (lower limb vs upper limb), the time from stroke onset (< 16 weeks vs ≥ 16 weeks), and the tDCS current density (≥ 0.05 mA/cm^2^ vs < 0.05 mA/cm^2^). The analysis by subgroups was not performed based on the follow-up period as in the previous protocol because all but one study had a short follow-up period equal to or less than 3 months. RevMan software was used for quantitative analysis. The quality of evidence was classified for each outcome as high, moderate, low, or very low following the Grades of Recommendation Assessment, Development and Evaluation (GRADE) method [28].

## Results

After the duplicates were removed, 445 articles were identified as eligible, and 389 were excluded after the titles and abstracts were read. Finally, after the full texts were read, 10 RCTs [29–38] that met the inclusion criteria were included in this systematic review and meta-analysis (Fig. 1). Additional information was requested from the authors of five studies [29, 31–34], but a response was received from only one author [34].

### Qualitative summary of the included studies

All included studies were sham controlled. The effect of active anodal tDCS was compared with those of sham tDCS and both therapies combined with robot-assisted rehabilitation. Of the included studies, seven were aimed at treating the upper limbs [30, 32, 33, 35–38], and three [29, 31, 34] were aimed at treating the lower limbs (Table 1). The sample size comprised 368 enrolled participants (n = 207 in active tDCS groups and n = 173 in sham tDCS groups); n = 159 were women (43.2%), n = 299 had ischaemic stroke (81.25%), n = 122 had cortical lesions (33.15%) and n = 60 had subcortical lesions (16.3%). The average age ranged between 52.7 and 75.25 years. The time since stroke onset was < 6 months (subacute stroke) in three studies [30, 35, 38], ≥ 6 months (chronic stroke) in five studies [29, 31, 34, 36, 37], and both subacute and chronic stroke were assessed in two studies [32, 33]. For these studies on two types of stroke, we contacted the authors to request the results of separate subacute and chronic analysis. The sociodemographic and clinical characteristics of the patients in the included studies are shown in Table 2.

In all included studies, tDCS was performed with anodal stimulation over the primary motor cortex (M1) from the stroke-affected hemisphere. Hesse et al. [30] included a third group in which a cathode over the M1 of the unaffected hemisphere was used as the active electrode in one of the three study arms. The cathode electrode was placed on the contralateral supraorbital area in all studies except in two studies [33, 36] where was applied over M1 of the unaffected hemisphere. In eight studies [30–32, 34–38], the tDCS session lasted for 20 min, and in the two remaining studies, the session lasted for 7[29] and 30 min [33]. tDCS was delivered simultaneously during RT (online stimulation) in six studies[29, 30, 32, 35, 36, 38]. The most common frequency of sessions was five sessions per weeks [29, 30, 33–35, 38]. The total number of sessions ranged from two to thirty-six. The electrode area was 35 cm^2^ in most of the studies [29, 30, 32–38], and only one study [31] used electrodes of 25 cm^2^. The current intensity ranged between 1 and 2 mA, and the current density ranged between 0.03 and 0.08 mA/cm^2^.

In the robot-assisted protocol, the duration of the session ranged from 20 to 60 min. The robots used for gait training were the Gait Trainer GT1 (Reha-Stim, Berlin, Germany) [29], Lokomat (Hocoma Inc, Switzerland) [31], and Walkbot (P&S Mechanics, Seoul, Republic of Korea) [33]. For upper limb therapy, the robot-assisted Bi-Manu Track (Reha-Stim Bi-Manu Track, Berlin, Germany) [30], Armeo® Spring (Hocoma AG, Switzerland) [32], REO Therapy System (Motorika, LTD, Israel) [33], InMotion wrist robot (Interactive Motion Technologies, Inc., Cambridge, MA, USA) [35], REAplan robot (Axinesis, Wavre, Belgique), MIT Manus (planar robot) [37] and InMotion WRIST robot (Bionik Laboratories Corp., Watertown, MA, USA) [38] were used (see Table 1).

The change in upper limb function was measured with the FM/ue scale in six studies [30, 32, 33, 35, 37, 38] and the Box & Block test in five studies [30, 33, 35, 36, 38]. The effect on lower limb function was analysed by the 10 MWT in three studies [29, 30, 34]. With regard to the secondary variables, six studies assessed strength by the MRC scale in upper [30, 37] and lower limbs [34] or MI scale in two studies for upper limbs [35, 38] and one study for lower limbs [29], four studies assessed spasticity by the MAS scale in the upper [30, 35, 38] and lower limbs [29], two studies [30, 37] assessed functional independence for upper limbs by the BI scale, and three studies [35, 36, 38] assessed upper limb velocity. Additionally, some studies assessed other variables and/or scales that were not within the objectives of the registered protocol of this review (see Table 1). The longest follow-up period was 6 months [37]. The follow-up period was less than or equal to three months (2 to 12 weeks) in five studies [29–32, 34], and in the remaining four studies [33, 35, 36, 38], there was no follow-up period (Table 1). Five studies reported lost to follow-up [30–32, 34, 36, 38]: a total of n = 30 (8.6%), with n = 17 from the experimental group (tDCS) and n = 13 from the control group Adverse effects and/or complications were specifically stated in nine of the ten included studies [29–33, 35–38]. Of these nine studies, five did not report any adverse events, and the other four [30, 32, 33, 37] reported mild adverse effects related to tDCS (Table 1).

### Risk of bias in the included studies

**Fig. 2:**
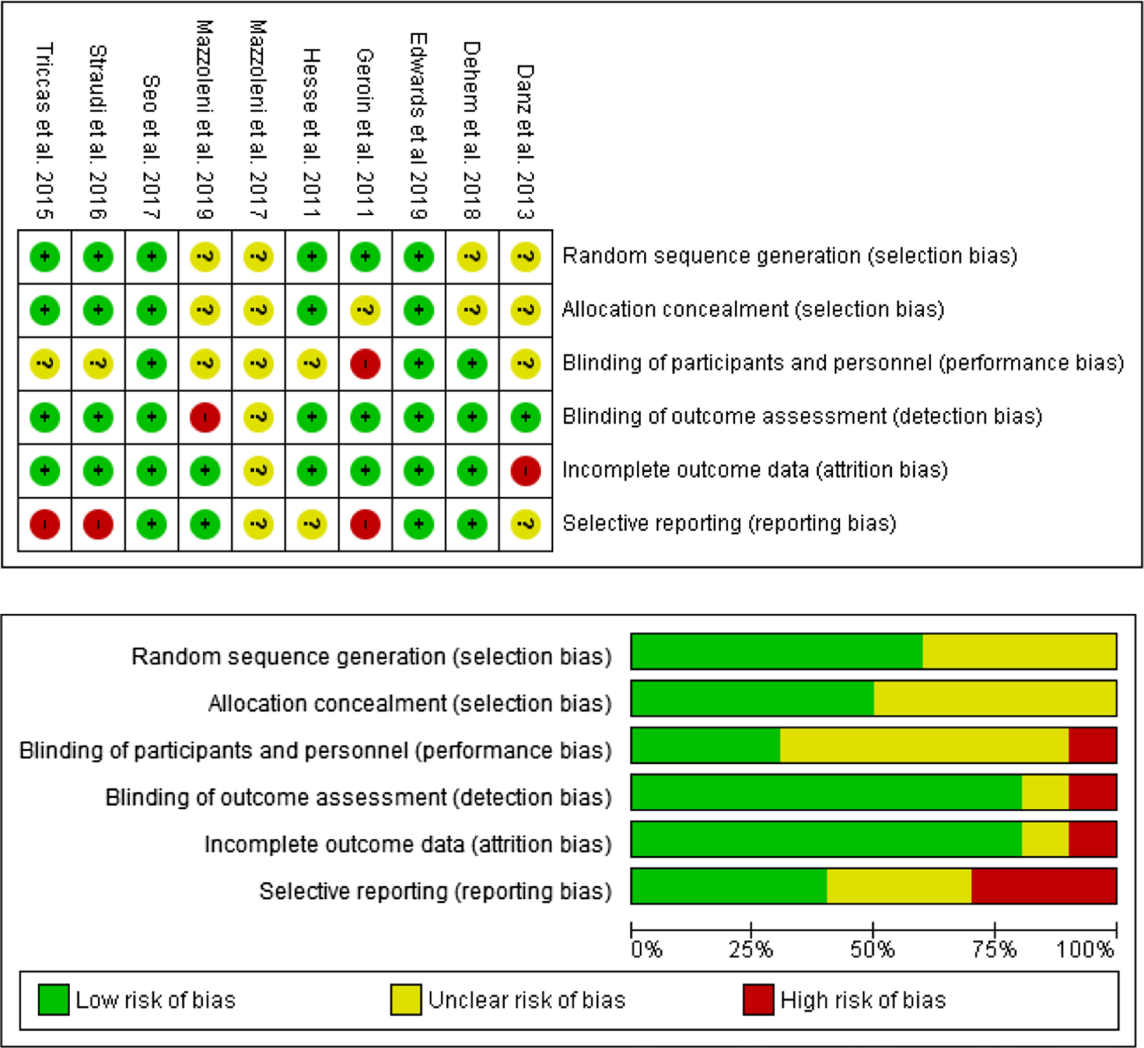
Risk of bias summary. Review authors' judgements about each risk of bias item for each included study (upper figure). Risk of bias item presented as percentages across all included studies (lower figure)

Figure 2 shows the risk of bias for the ten included studies. Four trials presented an unclear selection bias [31, 35, 36, 38] since the way in which the participants were randomized to groups was not described in detail. In terms of performance bias, six studies [30–33, 35, 38] had an unclear risk since the blinding of the participants but not the blinding of the personnel was possible. However, the study by Geroin et al. [29] was assessed as having a high risk of bias regarding the blinding of the participants and personnel, as the researchers kept the device turned off throughout the session in the sham group instead of using ramps at the beginning and end of the session. Eight of the ten assessed trials were rated as having a low risk of detection bias.

Only the two studies carried out by Mazzoleni et al. [35, 38] were rated as having an unclear and high risk of bias because the authors did not specify whether assessor blinding was conducted and because the study was single blinded, respectively. Regarding the outcome data, only the study published by Danz et al. [31] was considered to have a high risk of attrition bias, as the authors reported only the change scores for the main variable to measure the intervention effect. Four studies [35–38] were considered to have a low risk of reporting bias due to the protocols being previously registered, and three studies [30, 31, 35] were considered to have an unclear risk since the protocols had not been previously registered. Three studies were rated as having a high risk; Geroin et al. [29] did not report the results of the spasticity outcome, Triccas el al. [32] reported some secondary variables that were different from those registered in the previous protocol, and Straudi et al. [33] did not report spasticity or motor-evoked potential outcomes, although these outcomes were included in the previous protocol.

**Fig. 3:**
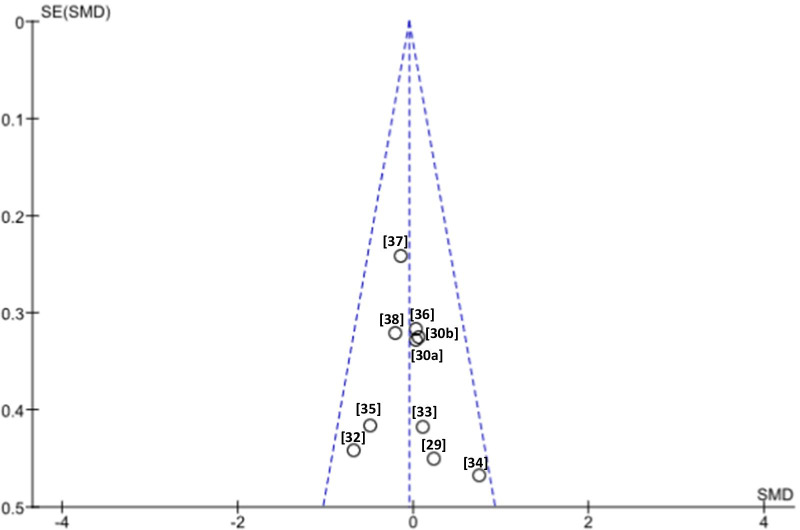
Funnel plot of comparison active tDCS + robotic rehabilitation Vs sham tDCS + robotic rehabilitation for the main outcome functionality. The references of the studies are shown in brackets. Asymmetries were not observed

The risk of publication bias was considered low since the distribution of the main variable (function) in the funnel plots was not asymmetrical (Fig. 3).

### Quantitative summary: effects of active tDCS versus sham tDCS both methods combined with robotic-assisted rehabilitation

According with the objective of this meta-analysis and the protocol published in PROSPERO, a quantitative analysis of the main variable function was performed. This effect was investigated in ten studies, seven for upper limbs [30, 32, 33, 35–38] and three for lower limbs [29, 31, 34]. The secondary outcome strength was investigated in six studies [29, 30, 35, 37, 38], the spasticity in three studies [30, 35, 38], the functional independence in two studies [30, 37], and the velocity of upper limb movements in three studies [35, 36, 38].

### Effect on function

**Fig. 4:**
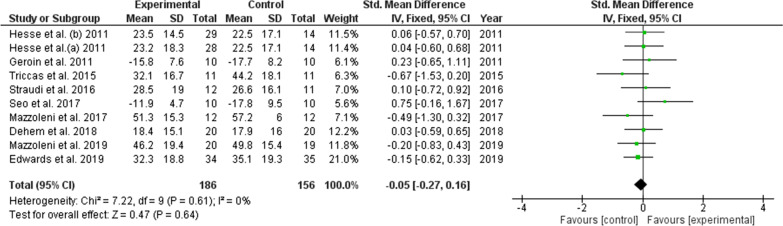
Forest plot of comparison between experimental group (active tDCS + robotic rehabilitation) and control group (sham tDCS + robotic rehabilitation) for the main outcome functionality. In Hesse et al. study (a) Anodic stimulation arm (b) Cathodic stimulation arm

Figure 4 summarizes the trials that assessed the effect of the combination of active tDCS and robotic-assisted rehabilitation compared with that of the combination of sham tDCS and robotic-assisted rehabilitation on function. The study by Danz et al. [31] was excluded from this analysis, as the authors reported the results as change scores. No differences were observed in the magnitude of improvement in function between the experimental group (active tDCS) and the control group (sham tDCS) (SMD = − 0.05; 95% CI: − 0.27–0.16), and there was a low level of heterogeneity (I2 = 0%, p = 0.61). In addition, no differences were observed between the experimental and control groups in the individual analysis of the included studies (Fig. 4).

In the subgroup analysis of function, a potential effect of the tDCS combined with robotic-assisted rehabilitation was observed in the lower limb function (SMD = 0.48; 95% CI: − 0.15–1.12), which was limited by only two studies. A non significant effect of the combined therapy was found in the upper limb function (SMD = − 0.12; 95% CI: − 0.35–0.11) (Table 2). When this effect was compared in people with chronic stroke (≥ 6 months) and with subacute stroke (< 6 months), no differences were found (Chi2 = 0.8, p = 0.36) (Table 2). For this analysis, the study by Straudi et al. [33] was excluded since the results for subacute and chronic patients were reported together. In addition, no differences were observed in the results between different dosages or current densities applied regarding the subgroups of ≥ 0.05 mA/cm^2^ and < 0.05 mA/cm^2^ (Chi^2^ = 0.0, p = 0.99) (Table 2). The quality of the evidence for this outcome according to the GRADE guidelines was moderate, considering a serious risk of bias as a factor that rating down.

### Effect on strength

**Fig. 5:**
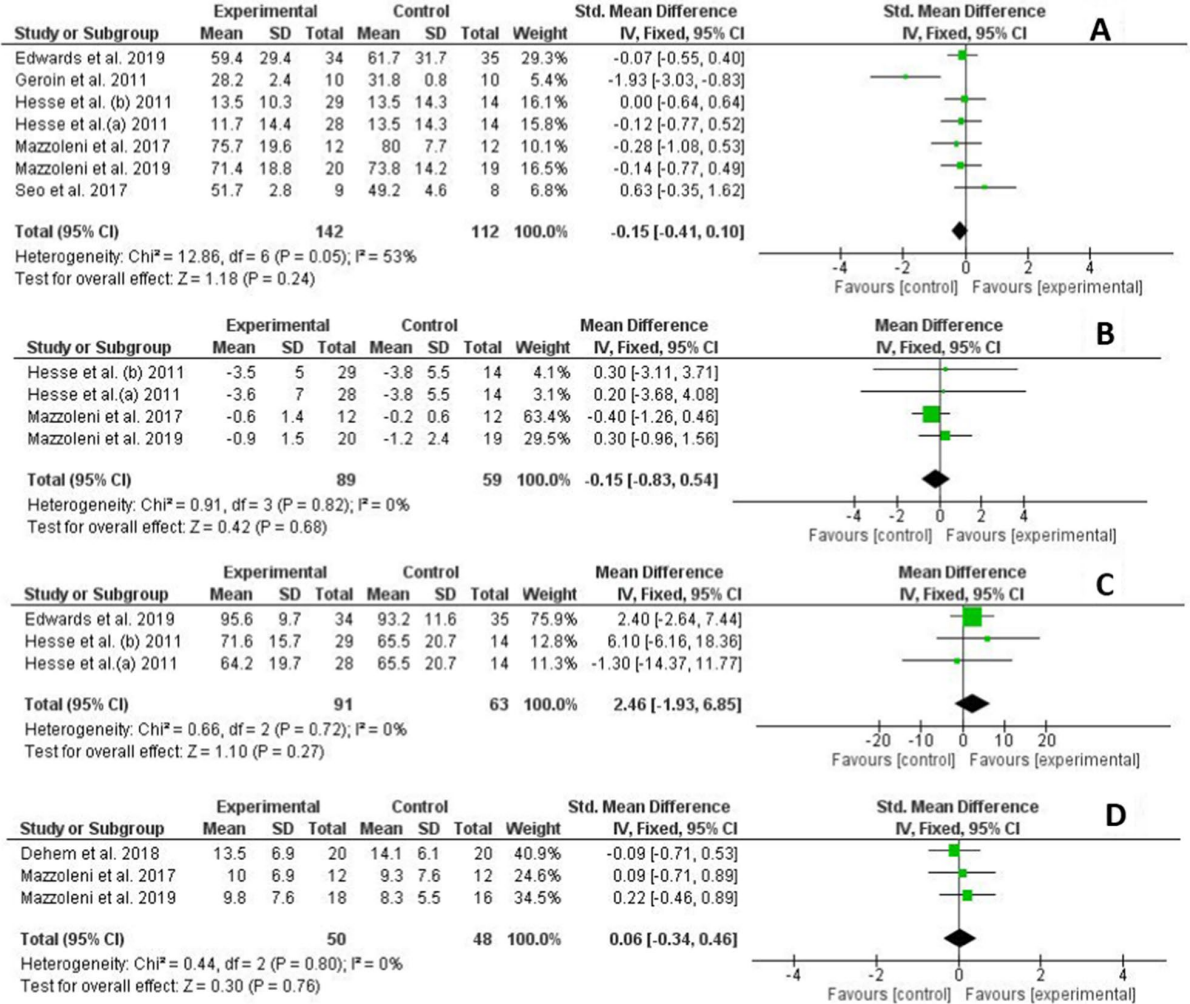
Forest plot of comparison between experimental group (active tDCS + robotic rehabilitation) and control group (sham tDCS + robotic rehabilitation) for secondary outcomes. **A** Effect on strength. **B** Effect on spasticity. **C** Effect on functional independence. **D** Effect on velocity of upper limb movements

Figure 5A summarizes the trials that assessed the effect of the interventions on strength. The overall strength score did not differ between the active and sham tDCS groups (SMD = − 0.15; CI 95%: − 0.4–0.1) and showed a moderate level of heterogeneity (I2 = 53%, p = 0.05). The individual results of the included studies showed that only Geroin et al. [29] reported differences between the active and sham groups favouring the sham group (Fig. 5). The quality of this evidence, according to the GRADE guidelines, was considered low, considering a serious risk of bias and heterogeneity of results as factors that rating down.

### Effect on spasticity

Figure 5B summarizes the trials that assessed the effect of the interventions on spasticity by the modified Ashworth scale. The overall effect on spasticity showed no differences between the active and sham tDCS groups (MD = − 0.15; 95% CI: − 0.8–0.5) and showed a low level of heterogeneity (I2 = 0%, p = 0.82). In addition, no differences were observed between the experimental and control groups in the individual results of the included studies (Fig. 5). The quality of this evidence, according to the GRADE guidelines, was moderate, considering a serious risk of bias as a factor that rating down.

### Effect on functional independence

Figure 5C summarizes the trials that assessed the effect of the interventions on functional independence by the Barthel Index. The overall effect on this outcome did not differ between the active tDCS and sham tDCS groups (MD = 2.5; 95% CI: − 1.9–6.9) and showed a low level of heterogeneity (I2 = 0%, p = 0.72). No differences were observed between the experimental and control groups in the individual results of the included studies (Fig. 5). The quality of this evidence, according to the GRADE guidelines, was moderate, considering a serious risk of bias as a factor that rating down.

### Effect on velocity of upper limb movements

Figure 5D summarizes the trials that assessed the effect of the interventions on upper limb movement velocity. The overall effect on this outcome did not differ between the active tDCS and sham tDCS groups (SMD = 0.06; 95% CI: − 0.3–0.5) and showed a low level of heterogeneity (I2 = 0%, p = 0.80). In addition, no differences were observed between the experimental and control groups in the individual results of the included studies (Fig. 5). The quality of this evidence, according to the GRADE guidelines, was moderate, considering a serious risk of bias as a factor that rating down.

### Adverse events and lost to follow

**Fig. 6:**
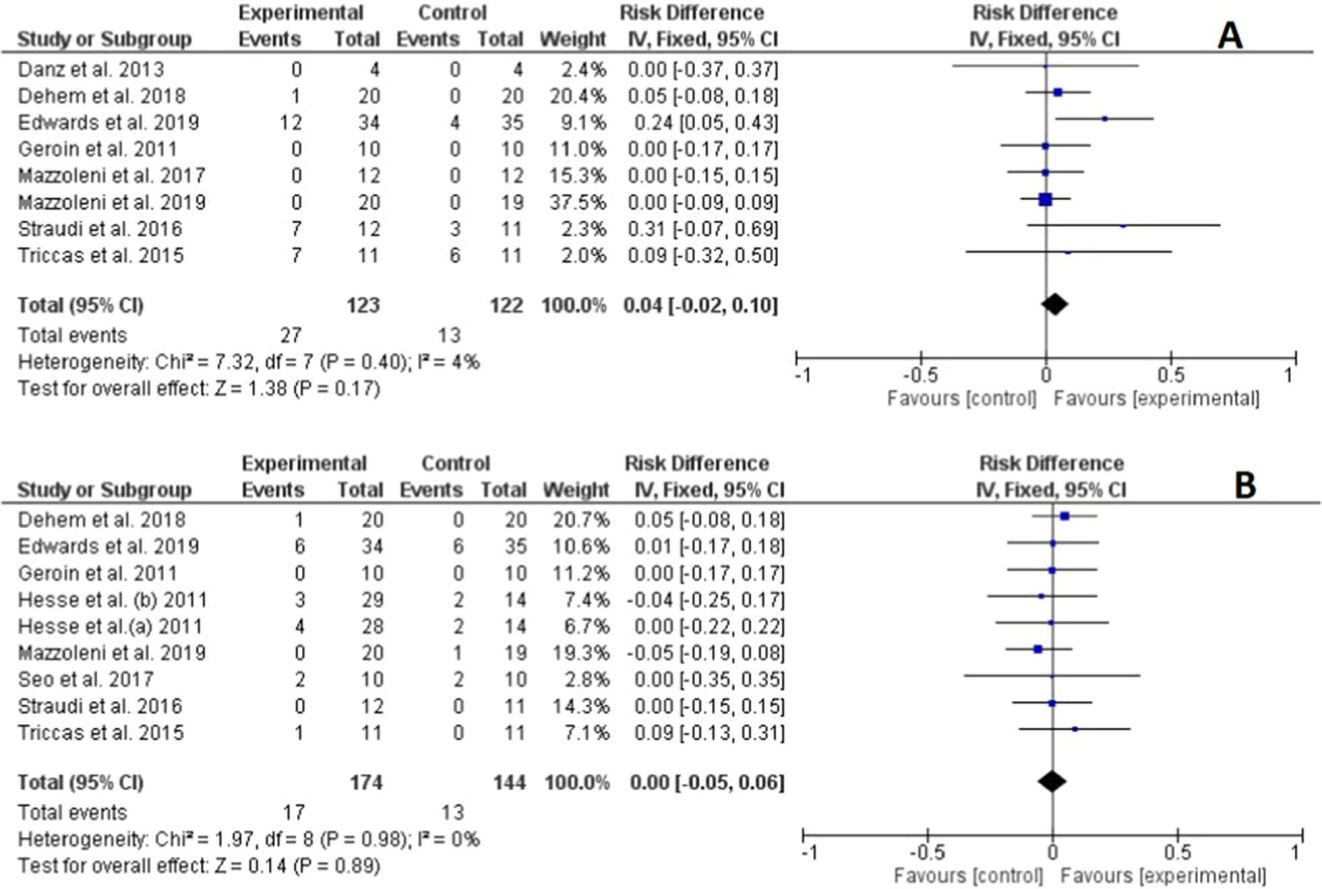
Forest plot of comparison between experimental group (active tDCS + robotic rehabilitation) and control group (sham tDCS + robotic rehabilitation) for adverse event (upper figure **A**) and lost to follow (lower figure **B**)

Figure 6 summarizes the trials that reported adverse events and the number of patients lost to follow-up. The overall analysis showed no risk difference for adverse events (RD = 0.04; 95% CI: − 0.02–0.1) and number of patients lost to follow-up (RD = 0.00; 95% CI: − 0.05–0.06) between the active tDCS and sham tDCS groups. Regarding the individual results of the included studies, only the study carried out by Edwards et al. [37] showed a high risk for adverse events in the active tDCS group. The quality of this evidence, according to the GRADE guidelines, was moderate, considering a serious risk of bias as a factor that rating down.

## Discussion

This systematic review and meta-analysis included 10 RCTs and 368 participants and was conducted to investigate the effects of tDCS as an adjunct to robotic therapy on limb function after stroke. In addition, the safety of tDCS and its effectiveness to improve strength, spasticity, functional independence and movement velocity were analysed. Currently, the evidence about the effectiveness of tDCS in previous systematic reviews and clinical trials is contradictory. The results obtained in the present meta-analysis showed non-significant improvement for the main variable (function) and secondary variables (strength, spasticity, functional independence and movement velocity), with a “moderate” to “low” recommendation level according to the GRADE guidelines. These results reveal that tDCS does not have an additional effect to RT alone on these outcomes.

A recently published guidelines and a meta-analysis on the use of tDCS for neurological and psychiatric disorders [39] found that when tDCS was combined with other therapies in the treatment of subacute and chronic stroke, patients showed improvements, and tDCS enhanced the effects of the adjuvant therapy. However, tDCS combined with intensive RT did not improve motor recovery to a greater extent than did RT. Our results are consistent with this guidelines, the subgroup analysis results showed no statistically significant differences between the experimental and the control tDCS groups, and both groups experienced improvements in clinical and kinematic variables.

Regarding other factors that may influence the effectiveness of tDCS, we can find the type and stage of the lesion, which can affect stroke evolution. A case-controlled study showed that when two patients had the same basal neurological severity, same functional disability, age and sex, haemorrhagic stroke patients had better prognosis than ischaemic ones [40]. In our review, of the 368 enrolled patients, 81.25% had ischaemic stroke, and most of the participants suffered from chronic-stage stroke and cortical lesions. Several studies have shown larger improvements with rehabilitation in the subacute stage (< 6 months) than in the chronic stage (> 6 months). These benefits may be related to spontaneous recovery, which is usually observed over third month after stroke onset. This period could be extended, depending on the severity, type and intensity of the intervention [32, 41, 42].

Factors including tDCS parameters, electrode size, electrode location, stimulation duration and the number of sessions could also affect the effectiveness of the intervention [43]. The tDCS protocols of the included studies in this review are too heterogeneous. The current density has been described as one of the main parameters that determine the effectiveness of tDCS. Two systematic reviews and meta-analyses showed a positive relationship between current density and the recovery of motor function. The current density determines the electrical field strength, which depends on the current intensity and electrode size. Higher current densities or smaller electrodes are associated with a higher efficacy of tDCS, which means a deeper penetration of the current into the scalp changing the excitability of the neurons under the electrode [19, 43]. Traditionally, intensities ranging between 1 and 2 mA are used in human studies. To date, the effects of current densities greater than 0.08 mA/cm^2^ are unknown.

Regarding adverse events, tDCS can be considered a safe therapy with some mild adverse effects observed in the included studies. It is necessary to perform studies where adverse events are actively assessed, as half of the included studies in this review did not report any adverse events, and one of the studies did not mention adverse events.

This systematic review and meta-analysis has limitations that could affect the obtained results: (1) due to differences in the patient characteristics, the study population is heterogeneous. The results obtained in this review cannot be generalized to haemorrhagic or subacute stroke patients because most of the enrolled participants had ischaemic or chronic stroke. (2) The sample sizes of the included studies were larger than 30 in only 3 studies [32, 39, 40]. This factor may influence the results, as studies with larger sample sizes, in which tDCS combined with other therapies, found significant differences in the variables analysed in stroke patients. (3) The heterogeneity in the tDCS parameters assessed made it difficult to compare the results. (4) There was variability in the number of sessions, the intervention protocol and the devices used for upper and lower limbs RT. (5)Most of the included studies had a short follow-up period, with only one study including a longer follow-up period of 6 months. (6) The study outcomes monitored differed across studies, which limited the ability to compare outcomes across studies.

## Conclusion

The reported findings suggest that the application of tDCS as an adjunct to RT does not enhance the effect of RT alone on upper limb function after stroke. This meta-analysis revealed that tDCS combined with RT may favour the lower limb function, however these results should be interpreted with caution because there were analysed by only two studies. Furthermore, positive results favouring tDCS combined with RT were not found in strength, spasticity, functional independence or movement velocity with an evidence confidence graded as “moderate”. Regarding adverse effects, tDCS can be considered a safe and well tolerated therapy with minor side effects. It is of relevance considering that most of the studies analysed were underpowered due to small sample sizes. It is also evident that the subject heterogeneity, the variability in the tDCS parameters and RT devices and the inconsistency of outcomes made difficult the comparison among studies. Further research studies should stratifying participants according to the type and stage of stroke including larger sample sizes, longer follow-up evaluation periods and adverse effects should be assessed to determine the optimal tDCS dose and parameters combined with RT.

## Supplementary Information

**Additional file 1.** Search strategy.

## Data Availability

Not applicable.

## Abbreviations

tDCS: Transcranial direct current stimulation
RT: Robotic therapy
RCTs: Randomized controlled trials
FM/ue: Fugl–Meyer motor assessment for upper extremities
10MWT: 10-Meter walking test
MI: Motricity Index
MRC: Medical Research Council Scale
MAS: Modified Ashworth Scale
BI: Functional independence with the Barthel Index
SMD: Standardized mean difference
CI: Confidence interval
MD: Mean difference
GBD: Global burden of disease
RD: Risk difference
MI: Motor cortex
