# Supplementary material for: Transcranial direct current stimulation combined with robotic therapy for upper and lower limb function after stroke: a systematic review and meta-analysis of randomized control trials
Source: J Neuroeng Rehabil. 2021 Sep 26;18:148. doi: 10.1186/s12984-021-00941-0 (PMC8474736; doi:10.1186/s12984-021-00941-0)
Supplement: Supplementary file 1 — Additional file 1. Search strategy. [file 12984_2021_941_MOESM1_ESM.docx]

1. **SEARCH STRATEGY**

- **PUBMED. ADVANCED SEARCH:**

**Nº OF SEARCHING AND FILE:**

1. (tDCS OR transcranial direct current stimulation) AND (robotic OR robot OR Lokomat OR exoskeleton [Title/abstract])= 2427 🡪 Filters “clinical trial” = 150
2. (Transcranial direct current stimulation [MeSH Terms]) AND (robotic [MeSH Terms]) =23
3. (Transcranial direct current stimulation) AND robotic [All fields]= 68 🡪 Filters “clinical trial”=25
4. (Transcranial direct current stimulation AND robotic) [Title/abstract] = 14
5. (tDCS AND robotic) [Title/abstract] = 12

- **PEDro. ADVANCED SEARCH:**

**Nº OF SEARCHING AND FILE:**

1. Abstract & Title: "transcranial direct current stimulation" AND "robotic". Therapy: Neurodevelopmental therapy, neurofacilitation. Problem/Body part: No appropriate value in this field. Subdiscipline: neurology. Topic: No appropriate value in this field. Method: clinical trial. Match all search terms (AND)= 0
2. Abstract & Title: “transcranial direct current stimulation” AND “exoskeleton”. Subdiscipline: neurology. Methods: clinical trial = 0
3. Abstract & Title: “transcranial direct current stimulation” AND “exoskeleton” = 0
4. Abstract & Title: “transcranial direct current stimulation” AND “robotic” = 0
5. Abstract & Title: “transcranial direct current stimulation” AND “robot” = 0
6. Abstract & Title: “transcranial direct current stimulation”. Method: clinical trial= 6
7. Abstract & Title: “electrical stimulation” AND robotic. Method: clinical trial= 4
8. Abstract & Title: “electrical stimulation” AND robot. Method: clinical trial= 4
9. Abstract & Title: “electrical stimulation” AND exoskeleton. Method: clinical trial= 4
10. Abstract & Title: “brain stimulation” AND robotic. Method: clinical trial = 0
11. Abstract & Title: “brain stimulation” AND robot. Method: clinical trial = 0
12. Abstract & Title: “brain stimulation” AND exoskeleton. Method: clinical trial = 0

- **COCHRANE LIBRARY. ADVANCED SEARCH:**

**Nº OF SEARCHING AND FILE:**

1. Title/abstract/keyword: ("transcranial direct current stimulation") OR (("tDCS") OR ("noninvasive brain stimulation")) AND ((robotic) OR (exoskeleton)) AND ((“stroke” OR “neurological disease”)). Filters: Clinical trial= 3593
2. Title: (transcranial direct current stimulation”) AND Title: (robotic) OR Title/abstract/keyword: (exoskeleton)=236
3. Title: (“transcranial direct current stimulation”) AND (Lokomat OR exoskeleton OR robot OR robotic)= 1
